# A Proposal For An Upgraded Microvolt T-wave Alternans Index With Consideration Of T-wave Amplitudes And The Rise In Heart Rate

**Published:** 2011-05-01

**Authors:** John E Madias

**Affiliations:** Mount Sinai School of Medicine of the New York University and the Division of Cardiology, Elmhurst Hospital Center, New York, NY

To the Editor:

Five years ago a conjecture was advanced that the magnitude of microvolt T-wave alternans (MTWA) was T-wave amplitude dependent [1], and thus it would be appropriate to index, adjust or correct the measured MTWA, regardless of any method used in its measurement, by the amplitude of the corresponding T-wave(s) used for its measurement[2,3]. In essence an index of MTWA (MTWAI) has been proposed [2,3]. It is also well appreciated that the magnitude of MTWA is heart rate-dependent; indeed often patients cannot mount the necessary rise in the heart rate during exercise stress testing, either due to the continuation of their beta-blocker therapy [4], or due to underlying chronotropic incompetence [5], or due to debility in cases of advanced class III, or IV heart failure, and thus their MTWA evaluation result is declared indeterminate. A strong recommendation for the continuation of maintenance of beta-blocker therapy in patients undergoing MTWA testing has been made in the past [3], and presently [4,5], and hopefully will be implemented as the standard approach henceforth. Nonetheless the previously proposed MTWAI [2,3] ignores the issue of heart rate (HR) dependence of MTWA amplitude, and this is something that this communication attempts to correct by proposing an upgrade of the MTWAI. The algorithm for such an upgrade will consist of the following steps: 1) The output of the MTWA metric, by any implemented method, in μV will be divided by the amplitude of the corresponding T-wave(s), used in the calculation of the MTWA, or any other conceivable mathematical attribute of the S-T segment, as previously proposed [1-3]. The resulting ratio will be in turn divided by the attained rise in HR, during the exercise testing, i.e., peak HR - resting HR. Such an improved MTWAI will correct for a possible chronotropic incompetence [5] or beta-blocker effect, in the assessment of MTWA. This should have utility if the magnitude (and not only the MTWA's mere presence, above the 1.9 μV threshold) is of diagnostic or prognostic value.

Although there is no doubt that MTWA amplitude is directly influenced by the rise in HR [6,7], the indexing proposed above may be simplistic, and should be considered only as food for thought, so that it might provide the impulse for prospective studies in this direction. Indeed there is no evidence for a linear relation of MTWA amplitude and HR, but the HR instead seems to exhibit a threshold behavior [6,7], with measurable MTWA above a certain HR and no MTWA below. Perhaps some attempt could be made to incorporate a non-linear standardization process. However this also may be prohibitively difficult, since the HR threshold over which MTWA appears is individual patient-specific, and the amplitude of MTWA is influenced by drugs, progression of disease [8], and varying levels of parasympathetic nervous system withdrawal and sympathetic nervous system arousal [9,10].  More work is needed to evaluate the dynamics of HR/MTWA in groups of patients. Perhaps the above proposed indexing could be considered in individual patients when they undergo serial MTWA, providing that all the other parameters which could influence the amplitude of MTWA are taken into consideration.
